# Deep Learning Classification of Unipolar Electrograms in Human Atrial Fibrillation: Application in Focal Source Mapping

**DOI:** 10.3389/fphys.2021.704122

**Published:** 2021-07-30

**Authors:** Shun Liao, Don Ragot, Sachin Nayyar, Adrian Suszko, Zhaolei Zhang, Bo Wang, Vijay S. Chauhan

**Affiliations:** ^1^Peter Munk Cardiac Centre, Division of Cardiology, Toronto General Hospital, University Health Network, Toronto, ON, Canada; ^2^Department of Computer Science, University of Toronto, Toronto, ON, Canada

**Keywords:** atrial fibrillation, unipolar electrogram, focal sources, machine learning, catheter ablation

## Abstract

Focal sources are potential targets for atrial fibrillation (AF) catheter ablation, but they can be time-consuming and challenging to identify when unipolar electrograms (EGM) are numerous and complex. Our aim was to apply deep learning (DL) to raw unipolar EGMs in order to automate putative focal sources detection. We included 78 patients from the Focal Source and Trigger (FaST) randomized controlled trial that evaluated the efficacy of adjunctive FaST ablation compared to pulmonary vein isolation alone in reducing AF recurrence. FaST sites were identified based on manual classification of sustained periodic unipolar QS EGMs over 5-s. All periodic unipolar EGMs were divided into training (*n* = 10,004) and testing cohorts (*n* = 3,180). DL was developed using residual convolutional neural network to discriminate between FaST and non-FaST. A gradient-based method was applied to interpret the DL model. DL classified FaST with a receiver operator characteristic area under curve of 0.904 ± 0.010 (cross-validation) and 0.923 ± 0.003 (testing). At a prespecified sensitivity of 90%, the specificity and accuracy were 81.9 and 82.5%, respectively, in detecting FaST. DL had similar performance (sensitivity 78%, specificity 89%) to that of FaST re-classification by cardiologists (sensitivity 78%, specificity 79%). The gradient-based interpretation demonstrated accurate tracking of unipolar QS complexes by select DL convolutional layers. In conclusion, our novel DL model trained on raw unipolar EGMs allowed automated and accurate classification of FaST sites. Performance was similar to FaST re-classification by cardiologists. Future application of DL to classify FaST may improve the efficiency of real-time focal source detection for targeted AF ablation therapy.

## Introduction

The pathogenesis of atrial fibrillation (AF) is complex, potentially involving localized drivers and abnormal atrial substrate outside the pulmonary veins (Heijman et al., 2016), which may account for the poor long-term success of pulmonary vein isolation (PVI) alone (Ganesan et al., 2013). Using panoramic high-resolution mapping, localized drivers, including focal electrical sources have been observed to sustain experimental AF (Lee et al., 2013, 2020), but their relevance in the pathogenesis of human AF is less clear. Detecting focal electrical sources in humans is challenging owing to the low spatial resolution of mapping techniques (Roney et al., 2017) and complex electrogram (EGM) features (DeBakker and Wittkampf, 2010).

To address these challenges, we have developed a pragmatic focal source detection algorithm, known as Focal Source and Trigger (FaST) mapping, where bipolar and unipolar EGMs are analyzed for periodicity and unipolar QS features as footprints of centrifugal wave propagation (Gizurarson et al., 2016; Kochhauser et al., 2017). In a randomized controlled trial, FaST sites were widely distributed in PV and extra-PV regions in all patients, and their ablation reduced AF recurrence compared to PVI alone (Chauhan et al., 2020; Nayyar et al., 2020). In FaST mapping, the accurate detection of sustained, periodic unipolar QS electrograms is critical and requires over reading by the cardiologist after the onset of the unipolar electrograms has been annotated by the FaST algorithm to guide morphology classification. This can be challenging when unipolar EGMs appear fractionated and non-stationary over 5-s recordings.

In this regard, machine learning, and more specifically deep learning (DL), has been used recently to automate classification of complex biomedical signals from ECG recordings (Hannun et al., 2019; Chang et al., 2021), but the utility of DL in raw EGM classification during AF has not been explored (Feeny et al., 2020). DL has the advantage of automatically learning features from raw signals without the need for *a priori* manual features engineering. We hypothesized that automating the detection of sustained, periodic unipolar QS EGMs using DL will improve the reliability and efficiency of FaST mapping for cardiologists performing AF driver catheter ablation. Our objective was to develop a DL model trained on raw unipolar EGMs to allow automated and accurate identification of FaST sites during AF as putative focal source targets for ablation.

## Materials and Methods

### Patient Population

The FaST randomized controlled trial evaluated the efficacy of FaST ablation as an adjunct to PVI in reducing AF recurrence compared to PVI alone in 80 patients with drug-refractory, high-burden paroxysmal or persistent AF (Chauhan et al., 2020). Real-time endocardial mapping of the left atrium (LA) during sustained AF was completed in 78 patients, who comprised the cohort for the present study. The study was approved by the University Health Network Research Ethics Board and all patients provided written, informed research consent.

### AF Mapping

The FaST mapping protocol and ablation outcomes have been previously described (Chauhan et al., 2020). Briefly, anti-arrhythmic drugs were held for 5 half-lives with the exception of amiodarone which was discontinued 1 month before mapping. LA mapping was performed during either spontaneous AF or induced AF using burst atrial pacing at CL 180–250 ms, and if necessary, intravenous isoprenaline (0.5–1 μg/min). Electroanatomic data was acquired using the CARTO^TM^ 3 (Biosense Webster, Diamond Bar, CA, United States) system and a roving 20-pole circular catheter (Lasso^TM^ Nav Variable, 15–25 mm diameter, 1 mm electrodes at 2–6–2 mm spacing, Biosense Webster, Diamond Bar, CA, United States). Stable catheter-tissue contact and signal quality were ensured before recording 5-s bipolar (bandpass 30–500 Hz) and unipolar EGMs (bandpass 0.05–500 Hz) at a sampling rate of 1,000 Hz. Unipolar EGMs were recorded only from one electrode of the bipolar electrode pair. All EGMs were exported for off-line analysis of FaST sites using custom software written in Matlab^TM^ (MathWorks Inc., Natick, MA, United States). Noisy EGMs with low signal:noise and EGMs recorded >5 mm from the LA endocardium were excluded to minimize far-field signal contamination.

### FaST Sites

**FIGURE 1:**
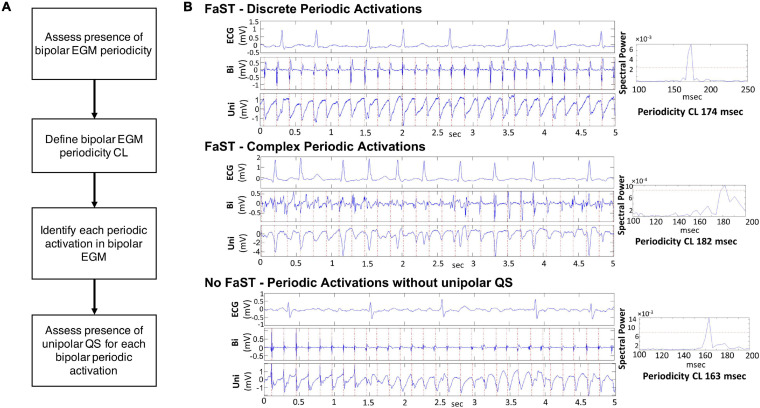
FaST Algorithm. **(A)** Hierarchical steps in FaST classification. **(B)** Representative examples of bipolar and unipolar EGMs from three different left atrial sites. In each example, the power spectral plot of the bipolar EGM indicates the presence of periodicity (i.e., spectral peak above threshold red horizontal line). The periodicity cycle length is calculated from the spectral peak (right panels). The first two examples are classified as FaST sites by the FaST algorithm based on the presence of bipolar EGM periodicity and sustained unipolar QS complexes for the duration of the 5-s recording. The top example shows discrete bipolar EGM complexes while the bottom example also contains complex, fractionated bipolar EGM complexes. In contrast, the third example is classified as No FaST because the unipolar EGM does not manifest sustained QS complexes for the duration of the 5-s recording, even though there is bipolar EGM periodicity. The first 1.4 s of the unipolar EGM manifests RS complexes, followed by QS complexes.

The hierarchical algorithm for FaST detection has been previously reported (Dalvi et al., 2016; Gizurarson et al., 2016; Chauhan et al., 2020; Nayyar et al., 2020) and is summarized in Figure 1. Briefly, each 5-s bipolar EGM underwent fast Fourier transformation after bandpass filtering (40–250 Hz followed by 0.5–20 Hz) and rectification. Periodicity was present if the spectral frequency with the largest spectral power contained at least 10% of the total spectral power. The corresponding periodicity CL was defined as the inverse of this frequency. Among bipolar EGMs demonstrating periodicity within a CL ranging from 100 to 250 ms (i.e., physiologic atrial refractory period), local bipolar periodic activations were annotated using a graph search function. For this purpose, candidate local activations were automatically selected provided their amplitude was above a noise threshold of 0.05 mV and a slew rate >0.014 mV/ms. Local periodic activations across the 5-s bipolar EGM were identified as those with the greatest number of consecutive candidate activations having the extracted periodicity CL, which satisfied the lowest cost of a matrix containing the difference between each candidate activation and the extracted periodicity CL (see Supplementary Methods) (Dalvi et al., 2016). This ensured that sustained periodic activations with predefined periodicity CL were identified regardless of their EGM amplitude, which itself is not a pre-requisite for defining local activation. These local periodic bipolar activations were then transposed to the corresponding unipolar EGMs in order to annotate unipolar EGM onset and thereby facilitate manual classification of unipolar morphology as QS or non-QS. FaST was defined based on the presence of sustained bipolar EGM periodicity and a dominant unipolar QS pattern (i.e., R/S ratio < 0.1) in >90% of EGMs over the 5-s recording, which was assigned manually by two cardiologists in real-time before ablation. Any disagreement in FaST classification by the cardiologists was resolved by consensus. FaST sites were classified as PV vs. extraPV and they were considered to be anatomically distinct if >7 mm from one another.

### Patient Cohorts and Data Augmentations

Patients were randomly divided into a training and testing cohort, and all periodic unipolar EGMs from both cohorts were firstly down-sampled to 200 Hz using fast Fourier transformation. Then, their magnitudes were normalized through a min-max feature scaling. To improve the generalizability of the model, four artificial data augmentations were implemented, namely baseline shifting, Gaussian noise, cropping and resampling. Baseline shifting added constant noise to the EGM signal, where the constant is sampled from a normal distribution. Gaussian noise added normal noise, sampled from a Gaussian distribution, to the EGM. Cropping randomly replaced a segment of data with zeros, while resampling further removed a data segment, but unsampled the shorter signal to the original length (Perez and Wang, 2017). A hyper-parameter was introduced to track the probability of augmentation and to ensure that both clean and noised examples were observed during training. The effectiveness of augmentation is demonstrated in the Supplementary Methods and Supplementary Figures 1, 2.

### One-Dimensional Residual Convolutional Neural Network

**FIGURE 2:**
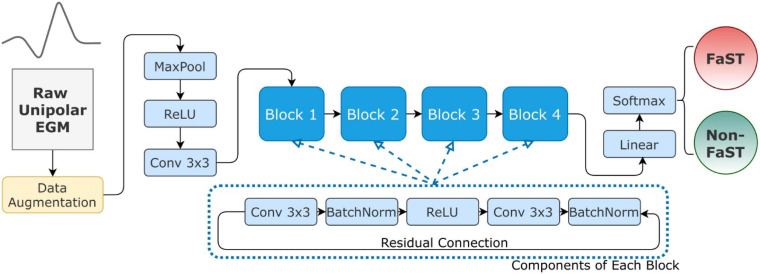
Convolutional Neural Network Architecture. The DL model inputs raw unipolar EGM **(right)** and outputs a binary decision as FaST vs. non-FaST **(left)**. For robust training, the raw inputs are normalized and augmented as described in the text. The architecture of the proposed network is inherited from ResNet-18, which has 4 residual blocks. Each block consists of 2 convolutional, 2 batch normalization, and one ReLU layers. Abbreviations: Conv – convolutional layer, MaxPool – 1-D max-pooling layer.

The DL model was designed to take the raw periodic unipolar EGM as input, and then output the probability of FaST on a continuous scale from 0 to 1. The model is a one-dimensional (1-D) residual, convolutional, deep neural network (CNN) which is implemented through PyTorch (Paszke et al., 2019). The network architecture is inspired by ResNet-18 for image recognition, which has been credible in a large number of datasets (He et al., 2016). In brief, it is an 18 layer neural network consisting of five residual convolutional blocks and one fully connected layer. Each block abstracts the features gradually from raw inputs to a higher level representation (LeCun et al., 2015). Specifically, each residual convolutional block consists of a convolutional layer, a pooling, a batch normalization, a dropout, a non-linear activation and a residual connection (LeCun et al., 2015). Notably, our EGM network replaces the 2-D convolution filters in each block of ResNet-18 by 1-D filters so that the architecture becomes suitable for unipolar EGM analysis. Our DL architecture is illustrated in Figure 2.

Due to similar structure, our DL model shares the same hyper-parameter settings with ResNet-18, such as kernel size, stride size and dropout rate (He et al., 2016). Although larger networks (e.g., ResNet-50, ResNet-101) and different architectures (e.g., EfficientNet) were also explored, we found ResNet-18 achieved the best testing performance in classifying FaST as shown in Supplementary Table 1. To prevent model overfitting, we searched a small subset of hyper-parameters, including batch size, initial learning rate and the learning rate scheduler. The best hyper-parameter combination was found through grid search with a three-fold cross-validation, which was then applied to the whole training cohort to train the DL model. The trained DL model was finally evaluated in the testing cohort. In terms of optimization details, the network is initialized by He-initialization and optimized by Adam (Kingma and Ba, 2014; He et al., 2015).

In addition, we investigated the performance of classic machine learning models to classify FaST, including logistic regression, support vector machine (SVM) and k nearest neighbor (KNN). Compared to DL, these classic models have a lower model complexity, which limits their ability to analyze complex data, such as EGMs. We reported the SVM and KNN with two different hyper-parameters, where the polynomial degree is either 3 or 10 for SVM, and the number of k neighbors is either 10 or 50 for KNN. These classic models were implemented through scikit-learn (Pedregosa et al., 2011).

### DL Model Discretization to Explain Classification

To explain DL classification as FaST vs. non-FaST, we adopted a gradient-weighted class activation mapping method (Guided Grad-CAM) to probe important features (Selvaraju et al., 2017). Grad-CAM is commonly used in computer vision to provide a contextual explanation for model decisions. Briefly, Grad-CAM defines the importance of a feature based on the changes in the classification output in response to a small variance or gradient in the feature. A larger change in output indicates that this feature is more important. For our study, Grad-CAM was applied because of similar architecture between our model and models in vision. Specifically, the gradient in the convolutional layer of the residual blocks of our model were probed. The importance of features was visualized as a 1-D importance plot where peaks indicated more importance.

### FaST Re-Classification by Cardiologists

Manual classification of FaST using the FaST algorithm at the time of PVI served as the gold standard. Subsequently, two cardiologists (VC, SN) independently performed blinded re-classification of periodic unipolar EGMs as FaST vs. non-FaST using the FaST algorithm in a subset of 100 EGMs, which included 50 random EGMs and 50 EGMs falsely classified by DL. The sensitivity and specificity of FaST re-classification by the cardiologists was evaluated relative to the gold standard. Inter- and intraobserver agreement among the cardiologists in FaST re-classification was assessed using the kappa statistic.

### Statistical Analysis

Continuous variables are presented as mean ± standard deviation. Comparison between patient cohorts was done using an unpaired *t*-test or Mann-Whitney *U* test where appropriate. Receiver operator characteristic (ROC) analysis was performed to evaluate the diagnostic performance of the DL algorithm for detecting FaST with results presented as area under the curve (AUC) and 95th percentile confidence interval (95% CI). Specificity was calculated at prespecified sensitivities of 85, 90, and 95% as well as the sensitivity of cardiologists re-classifying a subset of 50 random periodic unipolar EGMs. In order to complement ROC analysis for class-imbalanced datasets, the performance of DL was evaluated using the F1-score which is a harmonic mean of the positive predictive value and sensitivity (Saito and Rehmsmeier, 2015). A two-tailed *p*-value < 0.05 was considered statistically significant. Statistical analyses were performed using scikit-learn (Pedregosa et al., 2011).

## Results

### Patient and FaST Characteristics

Seventy-eight patients (age 61 ± 10 years, 74% males) were included with either high-burden paroxysmal AF (51%) or persistent AF (49%). The LA volume and LV ejection fraction were 44 ± 16 ml/m^2^ and 59 ± 8%, respectively (Table 1). Mapping was performed during spontaneous AF in 36 (46%) patients and after inducing sustained AF with programmed atrial stimulation in the remaining 42 (54%) patients. On average, 340 ± 60 LA sites from 60 ± 8 circular catheter acquisitions were analyzed per patient after excluding overlapping points and those with poor endocardial contact. FaST sites were identified in all patients (4.9 ± 1.9 per patient), including 2.1 ± 1.1 PV FaST and 2.8 ± 1.4 extra-PV FaST per patient.

**TABLE 1:** Baseline patient characteristics.

	**All patients (*n* = 78)**	**Training/validation cohort (*n* = 58)**	**Testing cohort (*n* = 20)**	***p*-value**
Age, years	61 ± 10	61 ± 10	59 ± 8	0.229
Male, n (%)	58 (74)	42 (72)	16 (80)	0.503
Body mass index, kg/m^2^	29 ± 5	30 ± 5	29 ± 5	0.598
LVEF,%	59 ± 8	58 ± 9	61 ± 4	0.097
**LA dimensions**				
LA diameter, mm	42 ± 7	42 ± 6	40 ± 8	0.383
LA volume, ml	90 ± 35	90 ± 33	91 ± 39	0.893
LA volume index, ml/m^2^	44 ± 16	43 ± 16	44 ± 16	0.811
**AF characteristics**				
High-burden paroxysmal, n (%)	40 (51)	29 (50)	11 (55)	0.700
Persistent, n (%)	38 (49)	29 (50)	9 (45)	0.700
Duration of AF, years	5.6 ± 5.0	5.9 ± 5.0	4.6 ± 3.4	0.245
**Comorbidities**				
Diabetes, n (%)	4 (5)	2 (3)	2 (10)	0.270
Hypertension, n (%)	37 (47)	25 (43)	12 (60)	0.192
Sleep apnea, n (%)	25 (32)	19 (33)	6 (30)	0.820
Obesity, n (%)	29 (37)	23 (40)	6 (30)	0.441
Coronary artery disease, n (%)	2 (3)	2 (3)	0 (0)	1.000
**Current antiarrhythmic drugs**				
Flecainide or propafenone, n (%)	29 (37)	26 (45)	3 (15)	0.017
Sotalol, n (%)	6 (8)	5 (9)	1 (5)	1.000
Amiodarone, n (%)	21 (27)	14 (24)	7 (35)	0.345
β-blocker, n (%)	37 (47)	28 (48)	9 (45)	0.800
Calcium channel blocker, n (%)	15 (19)	9 (16)	6 (30)	0.192
Number of failed AAD	1.7 ± 0.9	1.7 ± 1.0	1.6 ± 0.8	0.482

*AAD, antiarrhythmic drugs; CL, cycle length; LA, left atrium; LVEF, left ventricular ejection fraction; obesity–BMI > 30 kg/m^2^; renal dysfunction–eGFR < 50 ml/min/1.72 m^2^.*

### Performance of Deep Learning and Classic Machine Learning Models

**FIGURE 3:**
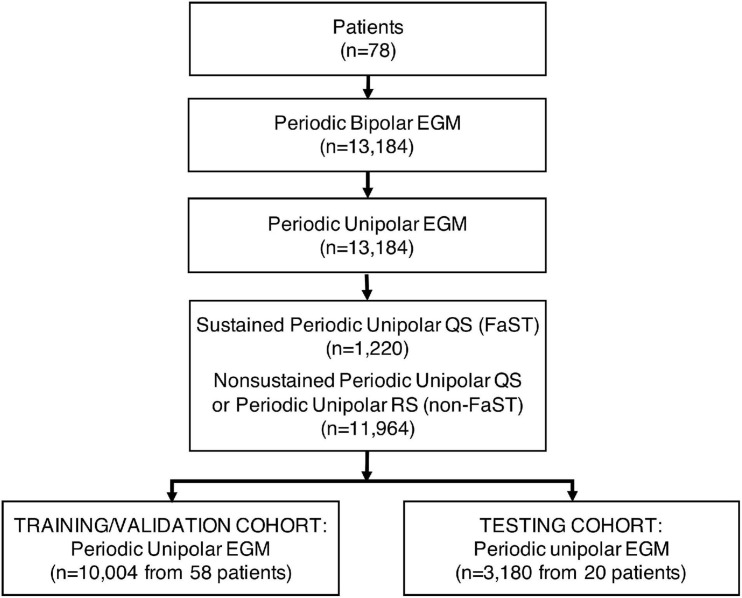
Consort Diagram for DL Training and Testing Cohorts. Patients (*n* = 78) were divided into a training/validation cohort (*n* = 58) and test cohort (*n* = 20). The number of periodic unipolar EGMs in each cohort is indicated.

**FIGURE 4:**
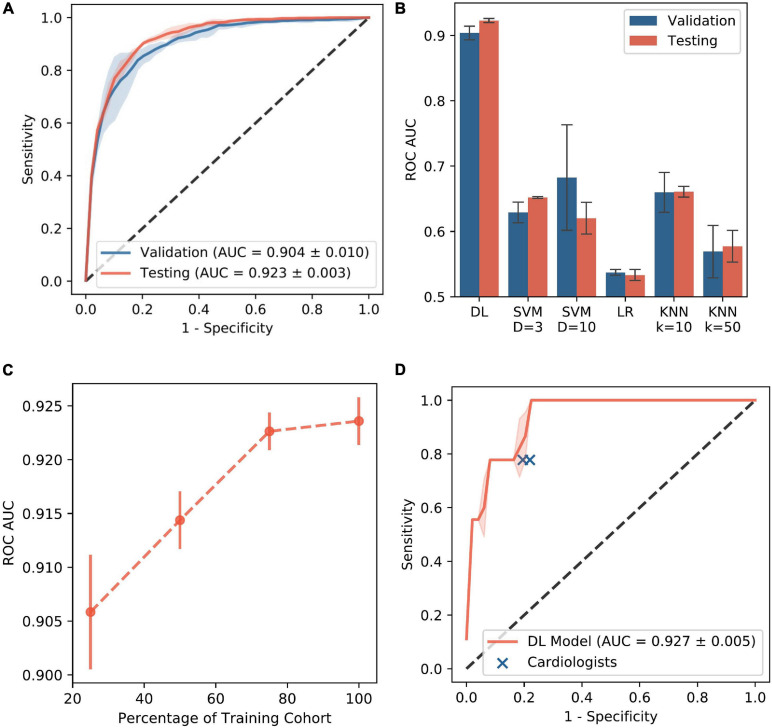
Performance of DL Model. **(A)** ROC curve is shown for FaST classification using DL model in the validation and testing cohorts. **(B)** ROC AUC is shown for DL and classic machine learning models. The error bars indicate standard deviation of different random seeds. **(C)** ROC AUC for DL is shown as a function of the training cohort size. **(D)** ROC curve is shown for FaST classification using DL model in a random sample of 50 periodic unipolar EGMs. The performance of two cardiologists for FaST re-classification is also plotted for comparison. AUC – area under curve; KNN – k nearest neighbor (either 10 or 50); LR – logistic regression; ROC – receiver operator characteristic; SVM – support vector machine (D refers to polynomial degree).

Among the 78 patients, a total of 13,184 periodic unpolar EGMs were recorded of which 1,220 (9.2%) had a dominant, sustained QS morphology (i.e., FaST) and the remaining 11,964 (90.7%) were non-FaST (Figure 3). The DL model was trained and validated using 10,004 periodic unipolar EGMs from a cohort of 58 patients, where the prevalence of FaST EGMs was 9.2%. Cross-validation in this cohort was achieved using five different random seeds, such that each seed produced a different validation cohort and a different network initialization (i.e., three-fold cross validation performed five times). The final DL model was then tested using 3,180 periodic unipolar EGMs from a testing cohort of 20 patients, where the prevalence of FaST EGMs was 9.4%. The clinical characteristics of the validation and testing cohorts were similar as shown in Table 1. The performance of DL in classifying FaST for the three-fold cross-validation and testing cohorts is demonstrated by the ROC curve in Figure 4A. The DL model achieved a high ROC AUC of 0.904 (95% CI 0.884, 0.924) and 0.923 (95% CI 0.917, 0.929) in cross-validation and testing cohorts, respectively. The AUC variance for the test cohort was < 0.5% demonstrating robustness of the DL model. In contrast, the performance of classic machine learning models, including logistic regression, SVM and KNN, was inferior to that of DL based on a lower ROC AUC, specificity and F1-score as shown in Figure 4B and Supplementary Table 2.

The performance of DL in classifying FaST was also evaluated using different prediction thresholds. Because the DL model has continuous output, ranging from 0 to 1, the prediction was classified as FaST when the DL output was above a threshold, which was based on achieving a predefined sensitivity of 85, 90, or 95% in detecting FaST. The respective specificity, positive predictive value (PPV), negative predictive value (NPV), F1-score and accuracy are shown in Table 2. DL had reasonably high specificity for each predefined sensitivity. In the case of 90% sensitivity, DL achieved a specificity of 81.9% (95% CI 81.8 – 82.0%), PPV of 33.6% (95% CI 33.3 – 33.9%), NPV of 98.5% (CI 95% 98.4 – 98.6%), F1-score of 0.486 (CI 95% 0.481 – 0.491), and an accuracy of 82.5% (95% CI 82.3, 82.6). Because DL performance improves with larger training datasets (LeCun et al., 2015), the performance of our DL model was further evaluated using smaller training cohorts. As shown in Figure 4C, the ROC AUC significantly improved when the test cohort size was increased from 25 to 75% of the original sample size. However, a further increase from 75 to 100% was associated with a marginal change in ROC AUC from 0.921 to 0.923, respectively, suggesting that our training cohort of 58 patients was adequately sized.

**TABLE 2:** Performance of DL model.

	**FaST Prevalence**	**Predefined Sensitivity**	**Specificity**	**PPV**	**NPV**	**F1-score**	**Accuracy**
Cross- Validation Cohort	9.2% (*n* = 1,220)	78*	87.3 (81.0 – 93.5)	40.0 (30.9 – 49.1)	97.4 (97.0 – 97.9)	0.528 (0.448 – 0.607)	86.4 (80.7 – 92.1)
		85	81.2 (75.9 – 86.6)	32.1 (28.7 – 35.6)	97.9 (97.6 – 98.3)	0.464 (0.429 – 0.499)	81.5 (76.8 – 86.2)
		90	73.7 (69.7 – 77.7)	26.3 (22.9 – 29.8)	98.5 (98.3 – 98.6)	0.406 (0.365 – 0.447)	75.2 (71.7 – 78.7)
		95	60.3 (54.9 – 65.7)	20.0 (18.8 – 21.2)	99.0 (98.7 – 99.3)	0.330 (0.313 – 0.347)	63.6 (58.9 – 68.2)
Testing Cohort	9.4% (*n* = 300)	78*	88.8 (87.4 – 90.3)	42.3 (39.1 – 45.5)	97.5 (97.5 – 97.6)	0.549 (0.522 – 0.576)	87.9 (86.5 – 89.2)
		85	85.0 (83.2 – 86.9)	36.7 (34.2 – 39.2)	98.0 (97.7 – 98.3)	0.509 (0.486 – 0.532)	84.9 (83.3 – 86.4)
		90	81.9 (81.8 – 82.0)	33.6 (33.3 – 33.9)	98.5 (98.4 – 98.6)	0.486 (0.481 – 0.491)	82.5 (82.3 – 82.6)
		95	68.7 (61.4 – 76.1)	24.1 (19.8 – 28.4)	99.1 (99.1 – 99.2)	0.383 (0.330 – 0.437)	71.1 (64.6 – 77.7)

*Sensitivity of cardiologist re-classifying FaST from a subset of 50 random periodic unipolar EGMs; NPV, negative predictive value; PPV, positive predictive value; 95% confidence intervals presented in parentheses.

### Performance of Deep Learning Compared to Re-Classification by Cardiologists

The reliability in FaST re-classification was evaluated in a random sample of 50 periodic unipolar EGMs from 18 patients by two cardiologists. In this 50 EGM subset, the proportion with FaST was modest at 18%. Intra- and interobserver variability was moderate based on a kappa of 0.43 and 0.46, respectively, but intraobserver variability improved (kappa 0.81) after the cardiologists reviewing their disagreements and retrained. Among these 50 EGMs, the DL model’s classification of FaST had an ROC AUC of 0.927 (95th CI 0.916, 0.938) (Figure 4D), which was similar to that of the whole periodic unipolar EGM dataset. In the subset of 50 random EGMs, the sensitivity and specificity in classifying FaST with DL was 78.1 (95th CI 77.6, 78.7) and 82.2 (95th CI 80.0, 84.4), respectively, which was similar to that of the cardiologists (sensitivity 77.8, specificity 79.0) (Figure 4D). Among the EGMs with interobserver agreement (*n* = 35 of 50), the DL model’s classification of FaST had a higher ROC AUC of 0.980 (95th CI 0.980, 0.986).

### Characterizing False Classifications by Deep Learning

**FIGURE 5:**
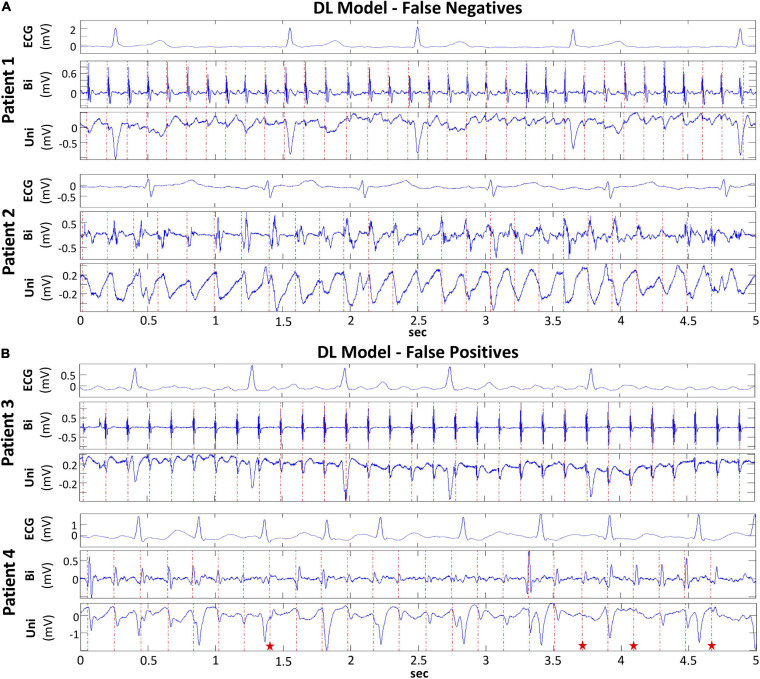
False Classification of FaST and non-FaST using DL Model. **(A)** False negative classification of FaST using DL due to low-amplitude, sustained periodic unipolar QS complexes near PV ostium (Patient 1, top panel) and broad, slurred unipolar QS complexes (Patient 2, bottom panel). **(B)** False positive classification of FaST using DL due to sustained unipolar rS complexes with small r waves (Patient 3, top panel) and near-sustained periodic unipolar QS complexes (red stars – rS complexes) (Patient 4, bottom panel).

In order to evaluate the basis for the false classification of FaST and non-FaST by DL, a subset of 50 periodic unipolar EGMs were selected, which comprised 25 false negative EGMs with the lowest DL predicted probability for FaST, and 25 false positive EGMs with the highest DL predicted probability for FaST. False positive classification by DL was commonly due to borderline EGMs with small rS complexes or non-sustained periodicity. In contrast, false negative cases by DL were most often the result of EGM fractionation or low amplitude/slewed QS complexes, such as near the PV ostium as shown in Figure 5. Given the complexity of these EGMs, the reliability in FaST re-classification was assessed by two cardiologists. In this 50 EGM subset, the proportion with FaST was 50%, which included all 25 false negative EGMs. Intra- and interobserver variability in FaST re-classification was poor based on a kappa of −0.08 and −0.02, respectively, which was concordant with the false classification or disagreement with DL. However, intraobserver agreement among the two cardiologists improved (kappa 0.71) after they reviewed disagreements and retrained.

### Discretization of Deep Learning to Explain FaST Classification

**FIGURE 6:**
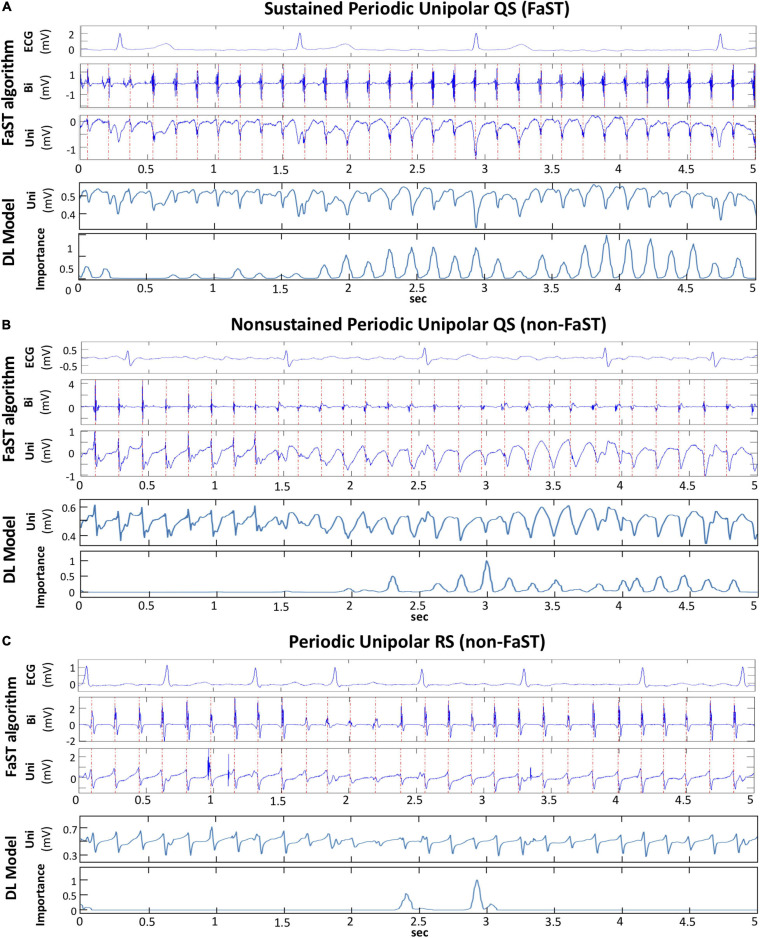
Discretization of DL model to explain classification. **(A)** FaST site defined by the FaST algorithm based on sustained, periodic unipolar QS for 5-s. Bipolar EGM and unipolar EGM are shown with dashed, red vertical lines annotating periodic activations. The DL model inputs the raw unipolar EGM without annotations and the importance plot from convolutional layer 3.0 demonstrates peaks corresponding to the majority of atrial unipolar QS complexes, but not the far-field ventricular complexes during the 5-s recording. **(B)** Non-FaST site defined by the FaST algorithm based on non-sustained periodic unipolar QS. The first 8 complexes are unipolar RS, while the rest are unipolar QS. The importance plot from the DL model’s convolutional layer 3.0 demonstrates peaks corresponding to the majority of atrial unipolar QS complexes, but not the atrial unipolar RS complexes. **(C)** Non-FaST site defined by the FaST algorithm based on the absence of unipolar QS complexes. Accordingly, the importance plot from the DL model’s convolutional layer 3.0 demonstrates virtually no peaks. There are 2 peaks which correspond to atrial unipolar rS complexes, similar in morphology to QS complexes.

From the subset of 100 periodic unipolar EGMs used above to evaluate observer reliability and false classification of FaST, a random sample of 10 EGMs were input into Grad-CAM in order to determine which convolutional layers of the DL model best tracked unipolar QS complexes. Our results suggest that Grad-CAM’s importance plot from convolutional layer 3 identified atrial unipolar QS complexes most consistently in all 10 EGMs. Figure 6 shows three examples of periodic unipolar EGMs from FaST and non-FaST sites where EGM onset is annotated with a vertical red line using the FaST algorithm. In each example, the importance plot from convolutional layer 3 demonstrates periodic peaks of importance that coincide temporally to most atrial unipolar QS complexes, while ignoring atrial unipolar RS complexes and far-field ventricular unipolar QS complexes. These importance plots provide a visual explanation of DL’s classification of FaST vs. non-FaST.

## Discussion

Our DL model automatically classified periodic unipolar EGMs with sustained QS complexes (i.e., FaST) during AF without the requisite for EGM segmentation or annotation. The DL model’s accuracy in FaST classification was 82.5% (ROC AUC of 92.3), which is high considering the low prevalence of FaST EGMs (9%) and the spatiotemporal variability in unipolar EGM morphologies. False detection of FaST was attributed to ambiguous, time-varying unipolar EGM signal features, but in these instances the reliability in re-classifying FaST was also poor among cardiologists, indicating that DL’s performance was on par with that of the cardiologists. For select EGMs, introspection of the DL convolutions identified the layer that tracked individual periodic unipolar QS EGMs, thereby providing visual verification of DL performance.

Focal sources are a well-established mechanism of AF, and have been demonstrated in a canine model of vagal AF (Lee et al., 2013, 2020) as well as in human AF (Lee et al., 2015, 2017). Using 512-electrode, high-density, biatrial activation mapping, Lee et al. (2015, 2017) demonstrated focal sources lasting up to 30 s during AF. At their epicenter, focal sources manifested fairly discrete, periodic unipolar QS EGMs. Our FaST algorithm searches for similar signal features to identify putative focal sources, but to improve specificity, unipolar QS periodicity must be sustained for 5-s. To avoid ambiguity in unipolar morphology classification, the onset of the unipolar EGM is annotated based on a graph search function whose input is the respective periodic bipolar EGM. However, unipolar QS classification is still performed manually and therefore susceptible to interpretation by the cardiologist, especially when morphology features are ambiguous, albeit periodic. This accounts for the moderate intra- and interobserver agreement in FaST re-classification in a random subset of periodic unipolar EGMs (kappa 0.43–0.47), and essentially no intra- or interobserver agreement in a subset falsely classified by DL. However, intraobserver agreement did improve (kappa 0.71–0.81) after cardiologists were retrained. These findings highlight the modest precision in the manual interpretation and classification of periodic unipolar QS EGMs during AF.

Despite this inherent limitation, DL achieved reasonable performance in classifying FaST based on an ROC AUC > 90% in the training and testing cohorts. This performance was similar when assessed in 75% of the training cohort indicating that data satisfaction was reached and that a larger training cohort would be unlikely to significantly improve classification accuracy. Based on ROC AUC, this performance was also comparable to re-classification by the cardiologists. False negative classification of FaST by DL was commonly due to fractionation at unipolar EGM onset and low amplitude/slew unipolar EGMs near the PV ostia. In false positive cases, periodic unipolar EGMs manifested small rS complexes or were non-sustained for only a few beats such that the prespecified criteria of >90% temporal stability for 5-s was not met.

### Comparison With Previous Machine Learning Studies

Deep learning has recently been applied to arrhythmia classification, but primarily in ECG recordings. Hannun et al. (2019) used residual CNN to classify a finite number of arrhythmias from a single-lead ECG strip, while Chang et al. (2021) employed the bi-directional long short term memory (LSTM) network to classify the same arrhythmias from a 12-lead ECG. To our knowledge, our study is the first application of DL to classify raw, intracardiac EGMs during AF. Similar to Hannun et al. (2019), we adopt residual CNN because all EGM signals were of the same duration, so that LSTM was not required. Machine learning models have also been developed to detect rotational activation during human AF, but the input training dataset was either color-coded phase maps (Alhusseini et al., 2020) or EGM frequency spectral features (Zolotarev et al., 2020) from a multielectrode array, and not raw EGMs as in our study. In the CNN model by Alhusseini et al. (2020), rotational activation was detected with an accuracy of 95%, while more classic machine learning models by Zolotarev et al. (2020) achieved an accuracy of 80–90% depending on size of the multi-electrode mapping array input into the model. In our study, the performance of classic machine learning models, such as logistic regression, SVM and KNN, in classifying FaST sites was inferior to that of DL, which highlights the computational proficiency of DL in EGM classification without the requisite for discrete feature input, such as unipolar EGM onset.

### Explainability of DL Model

Several techniques have been proposed to interpret machine learning classification in electrophysiology. We used Grad-CAM to evaluate explainability because the whole EGM signal is considered and the contribution of DL convolutional layers are weighted to generate visually interpretable importance plots (Selvaraju et al., 2017). Other approaches have been described, such as “occlusion mapping,” where portions of the signal are systematically deleted to assess the effect on DL performance (Bleijendaal et al., 2021), but this cannot be applied to our dataset because the entire 5-s EGM recording requires classified. Our findings with Grad-CAM suggested that the higher convolutional layers are more relevant in periodic unipolar QS classification, and in distinguishing atrial EGMs from far-field ventricular EGMs. These layers also detect the presence of sustained periodicity, which adds temporal dimensionality to the detection of individual unipolar QS complexes (Figure 6).

### Clinical Implications

Focal sources may be a relevant mechanism sustaining AF in some patients, which provides the rationale for accurate mapping. Given the complexity and non-stationarity of AF EGMs, automating focal source detection is difficult using multisite EGM recordings and conventional time-frequency domain analysis. Manual overreading may improve the robustness of focal source detection, but this is time-consuming and still susceptible to imprecision. In our randomized controlled trial, FaST sites were identified manually from an automated list of candidate periodic unipolar EGMs. FaST ablation terminated AF in 30% of patients, prolonged AF cycle length by 20 ± 14 ms among those with AF termination, and reduced AF recurrence by 48% at 1-year follow (Chauhan et al., 2020), suggesting that FaST sites defined with our non-DL FaST algorithm may identify focal sources. In the present study, FaST detection with DL using a training set of periodic unipolar EGMs was accurate, and the fully automated approach will ultimately improve interobserver variability and reduce FaST mapping time. As a clinical mapping tool, high sensitivity is important to identify the majority of putative focal sources, but equally important is the need to visually verify the EGM output so false positives are discarded. At a prespecified sensitivity of 90%, the specificity and accuracy of FaST detection with DL was high at 82 and 83%, respectively. Thus, DL has the potential to improve clinical AF mapping workflow by efficiently generating a comprehensive list of FaST sites, which can then be manually overread by the cardiologist. In addition, explainability of DL is essential to demystify the “black box” and facilitate adoption as a *bone fid* mapping tool in AF given the ambiguity of many EGMs and the uncertainty in their classification. Explainability was demonstrated with the importance plots using Grad-CAM for a subset of periodic unipolar EGMs in our study. Ultimately, our DL model may provide a more standardized approach to FaST detection as an adjunctive ablation strategy to PVI.

### Limitations

There are several limitations to acknowledge. First, FaST sites were defined based on a single recording site and not the activation pattern from a multielectrode array, but this was intentional to avoid the ambiguity of activation mapping in AF. Although unipolar QS are markers of focal sources, passive activation from epicardial-endocardial breakthrough or source-sink mismatch may also produce similar unipolar EGM morphology, but sustained periodicity would be unlikely. It is possible that DL training with neighboring unipolar EGMs from a multielectrode array will improve the specificity focal source detection (Zolotarev et al., 2020). Second, EGMs were rcorded for 5-s, but longer recordings could increase the sensitivity and specificity of FaST detection as putative AF sources. This was not performed to avoid circular catheter instability and poor EGM quality in some mapping region. Prior studies with ≥ 30 s recordings have been performed with a 64-electrode basket catheter, but this approach is disadvantaged by poor electrode-tissue contact and lower spatial resolution compared to FaST mapping. Third, our study population and periodic unipolar EGM dataset is relatively small and sourced from a single center. The performance of our DL model requires external validation in a larger patient cohort. The prevalence of FaST was also low, which can create class imbalance and a lower PPV and F1-score. To address this, we evaluated the performance of the DL model based on a number of predefined sensitivities and benchmarked this performance to cardiologists with good results as shown in Table 2 and Figure 4D. Fourth, our DL model, although comprehensive, has limitations in robustness and explainability, which are common to other DL algorithms (LeCun et al., 2015). Robustness was optimized by training the DL model on different sets of patients each with different random seeds, but this may still not be sufficient to address systematic noise (e.g., far-field ventricular EGM) or adversarial EGMs (e.g., borderline unipolar QS cases) (Papernot et al., 2016). For explainability, Grad-CAM was applied to probe the importance of features, but the analysis was qualitative because there are no clear metrics for quantitative benchmarking. Finally, DL was not used to guide real-time FaST ablation, however its reliability and efficiency will be evaluated in a future multicenter, randomized trial.

## Conclusion

Our novel DL model trained on raw unipolar EGMs in AF accurately identified FaST EGMs in patients with drug-refractory AF. Performance was similar to FaST re-classification by cardiologists. Explainability analysis showed that our DL model temporally tracked the hallmark periodic unipolar QS complexes that define FaST. DL is a promising computational tool to automate AF EGM classification and improve the efficiency of FaST detection, which may facilitate focal source mapping and ablation.

## Data Availability Statement

The raw data supporting the conclusions of this article will be made available by the authors upon reasonable request.

## Ethics Statement

The studies involving human participants were reviewed and approved by the University Health Network Research Ethics Board. The patients/participants provided their written informed consent to participate in this study.

## Author Contributions

SL: methodology design, data analysis, figure, and the manuscript preparation. DR: the figure preparation. SN: data analysis and critical review of manuscript. AS: data preparation. ZZ: critical review of the manuscript. BW: methodology design and critical review of the manuscript. VC: study conceptualization, methodology design, data analysis, the figure, and the manuscript preparation. All authors contributed to the article and approved the submitted version.

## Conflict of Interest

VC is the author of FaST mapping intellectual property (US 10,111,598 B2) owned by University Health Network, Toronto, ON, Canada. The study sponsors were not responsible for machine learning/mapping algorithm development, data collection, analysis or the manuscript preparation. The remaining authors declare that the research was conducted in the absence of any commercial or financial relationships that could be construed as a potential conflict of interest.

## Publisher’s Note

All claims expressed in this article are solely those of the authors and do not necessarily represent those of their affiliated organizations, or those of the publisher, the editors and the reviewers. Any product that may be evaluated in this article, or claim that may be made by its manufacturer, is not guaranteed or endorsed by the publisher.
