# Supplementary material for: Deep Learning Classification of Unipolar Electrograms in Human Atrial Fibrillation: Application in Focal Source Mapping
Source: Front Physiol. 2021 Jul 30;12:704122. doi: 10.3389/fphys.2021.704122 (PMC8360838; doi:10.3389/fphys.2021.704122)

**Supplemental Materials**

**Deep Learning Classification of Unipolar Electrograms in Human Atrial Fibrillation: Application in Focal Source Mapping**

**Running Title**:  Automated Focal Source Detection

Shun Liao MSc, et al.

**SUPPLEMENTAL METHODS**

**FaST algorithm - Assessment of periodic activations in bipolar EGM**

Several peak detection algorithms exist, but most have 2 important limitations. First, they assume all valid peaks to be peaks of interest and not specifically those that correspond to a periodic activity. Second, they assume true peaks are the largest local peaks. However, in AF recordings, neither assumption is valid; EGMs may be contaminated with aperiodic pulses, which, while genuine peaks, are not part of any sequence. Furthermore, many periodic peaks are not local maxima. These factors will compromise the ability of most methods to find peaks corresponding to a periodic cycle length.

In our approach, we identify periodic peaks in bipolar EGMs with both periodic and aperiodic components. Briefly the method works as follows:

1. Find the dominant periodicity in the signal (and the corresponding CL):

We employ the widely adopted dominant frequency (DF) analysis. In DF analysis, the signal is first filtered to enhance the dominant sinusoidal waveform. Then, a Fast Fourier Transform is performed on the processed signal and the frequency with the highest amplitude in its power spectral density distribution is considered the ‘dominant’ frequency.

1. Find the peaks corresponding to the dominant periodicity CL using Dijkstra's shortest cost path algorithm as follows:
2. We correct the DC shift in the given signal S and then find the set (Peak_EGM_) of all peaks in S whose gradient is greater than a threshold τ and whose absolute amplitude is greater than ρ. We then calculate the distance matrix, i.e., the matrix of the absolute values of the differences of all peak locations with respect to each other. Thus,

${Peak}_{Distances}= \left[ \begin{matrix} \left| P\left( 1 \right)-P\left( 1 \right) \right| & \cdots& \left| P\left( 1 \right)-P\left( N \right) \right| \\ \vdots& \ddots& \vdots\\ \left| P\left( N \right)-P\left( 1 \right) \right| & \cdots& \left| P\left( N \right)-P\left( N \right) \right| \end{matrix} \right]$ $\ldots(1)$

where P(N) refers to the Nth peak location in Peak_EGM_

1. From this matrix, we compute the cost matrix which is Peak_Cost_ = Peak_Dist_ – CL. Thus,

${Peak}_{Cost}=\left[ \begin{matrix} \left| {Peak}_{Dist}\left( 1,1 \right)-\mathrm{CL} \right| & \cdots& \left| {Peak}_{Dist}\left( 1,N \right)-\mathrm{CL} \right| \\ \vdots& \ddots& \vdots\\ \left| {Peak}_{Dist}\left( 1,N \right)-\mathrm{CL} \right| & \cdots& \left| {Peak}_{Dist}\left( N,N \right)-\mathrm{CL} \right| \end{matrix} \right]$ $\ldots(2)$ where ${Peak}_{Dist}\left( n,m \right)$ is the distance from node n to node m in Peak_EGM_

1. We then apply Dijkstra’s shortest path algorithm on Peak_Cost_ to find the shortest cost path between the first and last peaks in Peak_EGM_ and find the set of peaks (P’) corresponding to that path. The rationale behind doing so is that the costs in Peak_Cost_ indicate how much the distance between any two peaks differs from the CL. Hence, the shortest cost path will be the one that returns a set of peaks (P’) which are as periodic as possible (with a periodicity=CL).
2. While P’ will mainly contain peaks corresponding to the periodic CL, it may also contain some that do not which were selected to obtain a better cost. To remove the latter, only those peaks in P’ that are within 10% of CL from another peak are included in the final set of peaks (P). This ensures that P contains peaks with periodicity CL (i.e. their distance from their nearest neighbour is within a tolerance band of CL).
3. Since the first and last peaks in Peak_EGM_ may not be part of the periodic sequence, we re-implement Steps C and D considering the first n peaks in Peak_EGM_ as starting peaks and the last n peaks in Peak_EGM_ as ending peaks. Using n=3, we finally choose the final set of points (Ψ) for those starting/ending peaks whose cost for P is least.

**EGM Data Augmentation**

To improve the generalizability of the DL model, we proposed 4 different augmentation methods for the raw unipolar EGMs, namely baseline shift, Gaussian noise, cropping and resampling. Data augmentation in data analysis are techniques used to increase the amount of data by adding slightly modified copies of already existing data or newly created synthetic data from existing data.^1,2^ In our case, these augmentation methods add artificial noise into normalized EGMs to prevent the overfitting of DL model.

Regarding the detail of each method, “baseline shift” adds a constant to the EGM and the constant is randomly sampled from a normal distribution. “Gaussian noise” inserts small Gaussian noise (standard deviation 0.01) into the EGM which is significantly smaller than the standard deviation (>0.5) of the EGM signal. “Cropping” replaces a segment of EGM with zeros where the start of the segment and its length are randomly sampled from a uniform distribution. Finally, “resampling” resamples the EGM into a lower frequency and pads zeros at the end of the signal. The padding is to maintain the resampled sequence of the same length. To ensure that the raw EGM is observed by DL model, we introduce a probability to control how often these augmentations occur and this probability is set to 0.5. The four augmentations are illustrated in **Supplemental Figure 1**.

To evaluate the effectiveness of augmentation, we compared the DL model’s performance trained with and without augmented data. For this purpose, the same training scheme was implemented as described in the Methods. As shown in **Supplemental Figure 2**, the DL model trained without augmentation had an ROC AUC of 0.868, which was less than that of the DL model trained with augmentation (0.923).

**REFERNCES**

1. Shorten C, Khoshgoftaar TM. A survey on image data augmentation for deep learning. Journal of Big Data. 2019; 6(1):1-48.
2. Perez L, Wang J. The effectiveness of data augmentation in image classification using deep learning. *arXiv:1712 04621* 2017;Dec 13:[e-print].

**SUPPLEMENTAL RESULTS**

**Supplemental Table 1: Performance of Various CNN Architectures in Detecting FaST**

| **Methods** | **AUC** | **Predefined Sensitivity*** | **Specificity** | **F1-score** |
| --- | --- | --- | --- | --- |
| ResNet-18 | 0.923  (0.917 – 0.929) | 78^*^ | 88.8  (87.4 - 90.3) | 0.549  (0.522 – 0.576) |
| ResNet-50 | 0.915  (0.905 – 0.925) | 78 | 88.0  (86.6 - 89.4) | 0.529  (0.500 – 0.558) |
| ResNet-101 | 0.907  (0.892 – 0.923) | 78 | 87.5  (85.5 - 89.5) | 0.501  (0.475 – 0.528) |
| EfficientNet-B0 | 0.919  (0.913 – 0.925) | 78 | 88.3  (86.9 - 89.7) | 0.535  (0.512 – 0.558) |
| EfficientNet-B1 | 0.913  (0.901 – 0.925) | 78 | 87.9  (86.3 - 89.5) | 0.520  (0.501 – 0.539) |

*Benchmark sensitivity achieved by cardiologist re-classifying FaST in 50 randomly selected EGMs; AUC-area under curve; CNN-convolutional neural network

**Supplemental Table 2: Performance of Various Supervised ML models in Detecting FaST**

| **Methods** | **AUC** | **Predefined Sensitivity*** | **Specificity** | **F1-score** |
| --- | --- | --- | --- | --- |
| DL | 0.923  (0.917 – 0.929) | 78^*^ | 88.8  (87.4 - 90.3) | 0.549  (0.522 – 0.576) |
| SVM  D=3 | 0.652  (0.649 – 0.655) | 78 | 33.5  (33.2 - 33.8) | 0.187  (0.156 – 0.218) |
| SVM  D=10 | 0.620  (0.572 – 0.669) | 78 | 30.5  (27.4 - 33.6) | 0.169  (0.135 – 0.204) |
| Logistic regression | 0.533  (0.517 – 0.550) | 78 | 26.3  (25.1 - 27.5) | 0.121  (0.091 – 0.151) |
| KNN  k=10 | 0.661  (0.644 – 0.677) | 78 | 35.1  (33.3 - 36.9) | 0.211  (0.191 – 0.231) |
| KNN  k=50 | 0.577  (0.529 – 0.626) | 78 | 28.3  (25.5 - 30.1) | 0.135  (0.103 – 0.167) |

*Benchmark sensitivity achieved by cardiologist re-classifying FaST in 50 randomly

selected EGMs; AUC-area under curve; KNN-k nearest neighbors; ML-machine

learning; SVM-support vector machine

**SUPPLEMENTAL FIGURE LEGEND**

**Supplemental Figure 1: Augmentation Methods**

The EGM preprocessing (normalization) and data augmentation is illustrated in the following plots. All EGMs are normalized before training, and these 4 data augmentations occur by a probability during training.


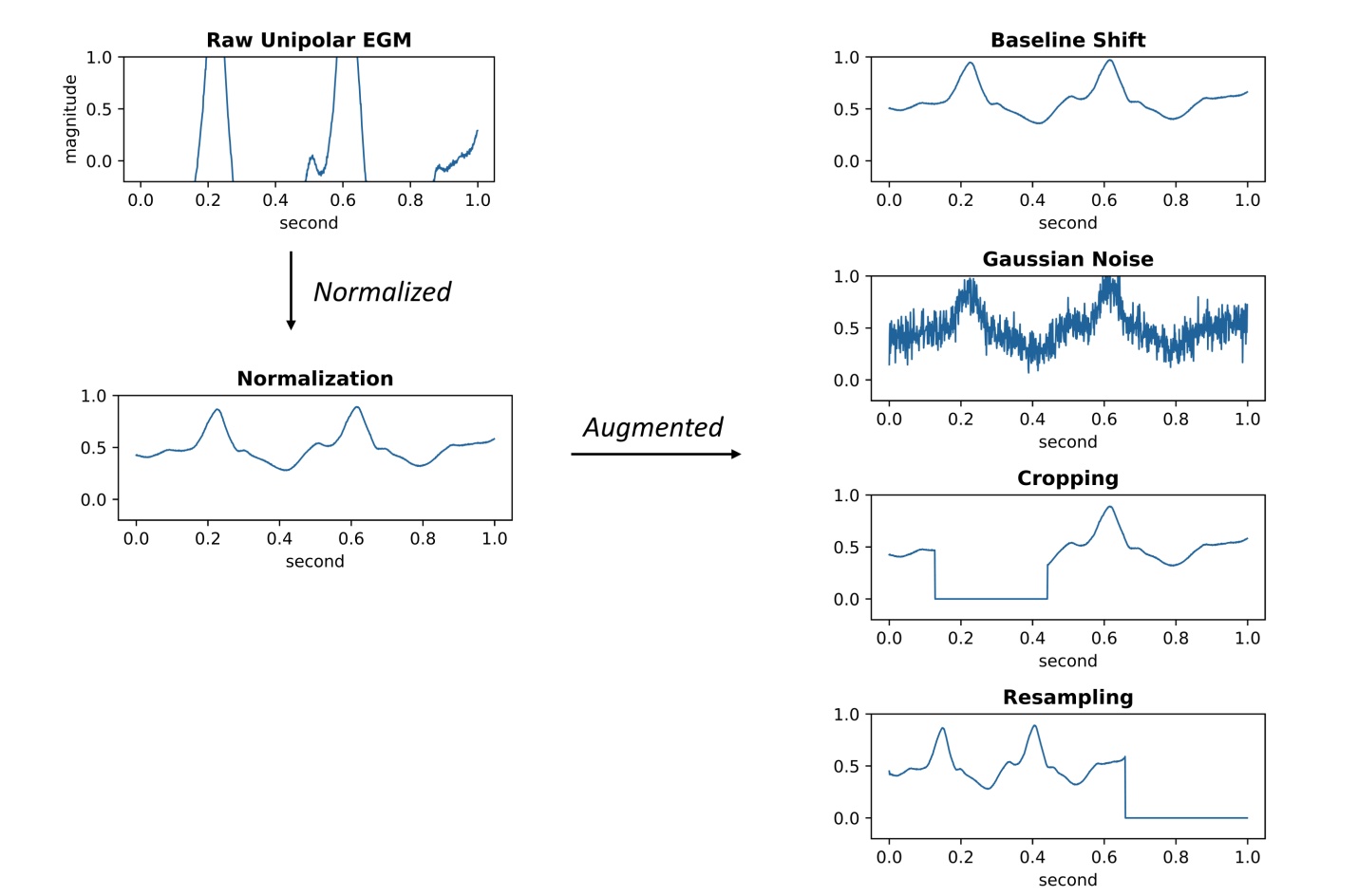


**Supplemental Figure 2 Ablation Result of Augmentation.**

The performance of the DL model is compared when trained with and without augmentation. The ROC AUC is higher with augmentation than without augmentation.


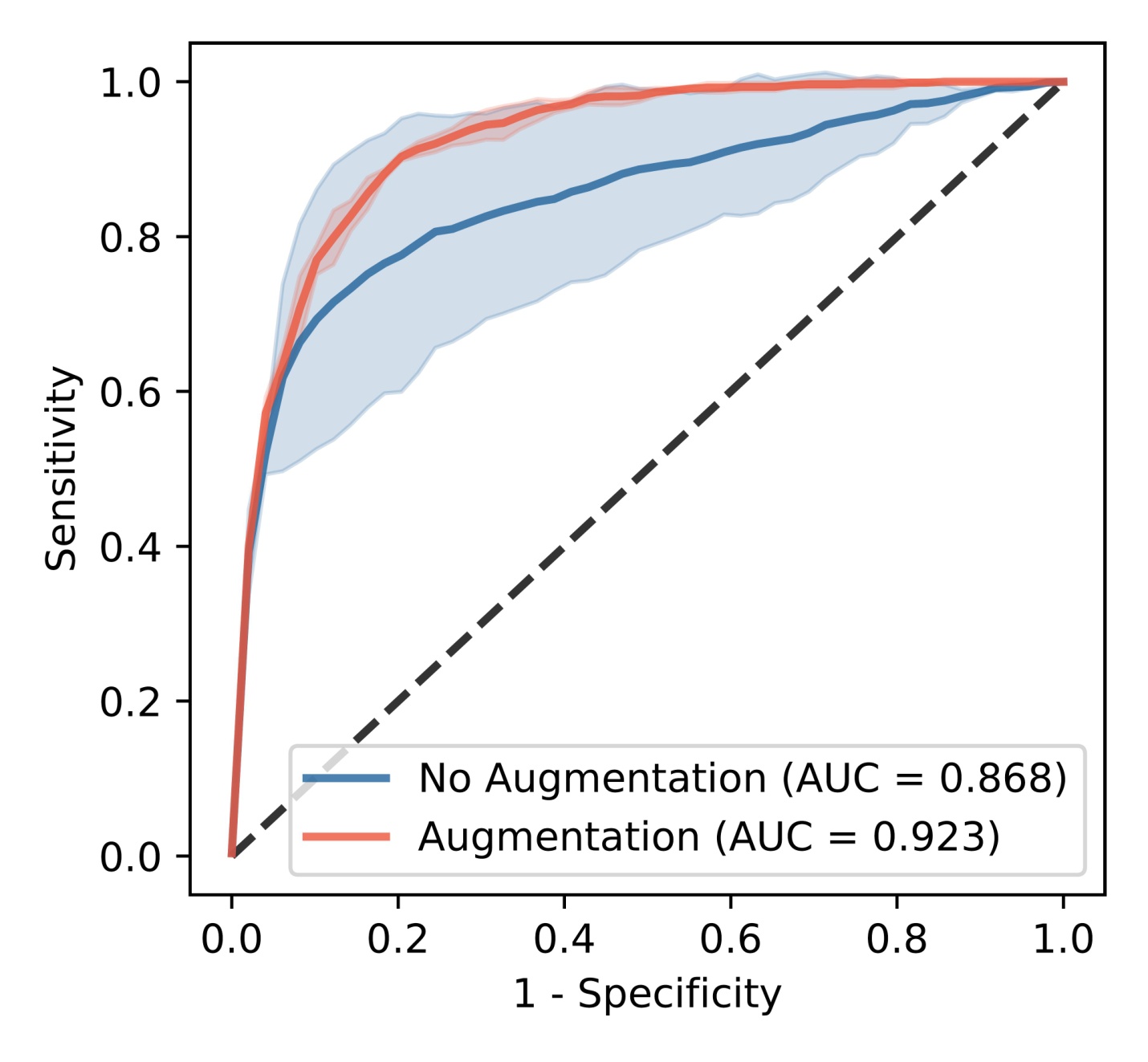

Supplement: Supplementary file 1 [file Data_Sheet_1.docx]
